# Supplementary material for: Selection of Non-Saccharomyces Wine Yeasts for the Production of Leavened Doughs
Source: Microorganisms. 2022 Sep 15;10(9):1849. doi: 10.3390/microorganisms10091849 (PMC9501029; doi:10.3390/microorganisms10091849)
Supplement: Supplementary file 1 [file microorganisms-10-01849-s001.zip › microorganisms-1879041-supplementary.pdf]

**Table S1.** Identification, based on blast comparison in GenBank of ribosomal region 26S rRNA of 10 yeast strains selected on the basis of RAPD-PCR cluster analysis.

| Cluster | Strain | Size | Closest Relative | % Identity | Sequence ID * |
|---|---|---|---|---|---|
| A | YFi12 | 573 | *Saccharomyces cerevisiae* isolate 20170721 | 100% | MF521980 |
| B | YG7 | 573 | *Torulaspora delbrueckii* YMTT5-1 | 100% | LC387296 |
| C | YC15 | 590 | *Pichia kudriavzevii* strain LY49 | 100% | KY705008 |
| D | YG8 | 576 | *Hanseniaspora uvarum* isolate 20170728 | 98% | MF521987 |
| E | YA17 | 578 | *Hanseniaspora uvarum* isolate SFM28 | 100% | MG017569 |
| F | YC18 | 588 | *Hanseniaspora uvarum* isolate 11-1288 la | 100% | MH468682 |
| G | YA27 | 610 | *Saccharomyces cerevisiae* strain LY183 | 100% | KY705009 |
| H | YFa31 | 833 | *Metschnikowia pulcherrima* culture CBS:2243 | 98% | KY108490 |
| I | YA28 | 596 | *Hanseniaspora uvarum* isolate 11-1288 la | 100% | MH468682 |
| L | YS20 | 921 | *Zygotorulaspora florentina* CBS 746 | 99% | NG_058449 |

* Accession number of the sequence of the closest blast search.
